# Investigating brain uptake of a non-targeting monoclonal antibody after intravenous and intracerebroventricular administration

**DOI:** 10.3389/fphar.2022.958543

**Published:** 2022-08-29

**Authors:** Arthur J. Van De Vyver, Antje-Christine Walz, Mariette S. Heins, Afsaneh Abdolzade-Bavil, Thomas E. Kraft, Inja Waldhauer, Michael B. Otteneder

**Affiliations:** ^1^ Roche Pharma Research and Early Development, Pharmaceutical Sciences, Roche Innovation Center Basel, Basel, Switzerland; ^2^ Charles River Laboratories Den Bosch, Groningen, Netherlands; ^3^ Roche Pharma Research and Early Development, Pharmaceutical Sciences, Roche Innovation Center Munich, Penzberg, Germany; ^4^ Roche Pharma Research and Early Development (pRED), Roche Innovation Center Zurich (RICZ), Schlieren, Switzerland

**Keywords:** monoclonal antibody, pharmacokinetics, microdialysis, brain uptake, physiologically–based pharmacokinetic model, blood-brain barrier (BBB), blood CSF barrier (BCSFB)

## Abstract

Monoclonal antibodies play an important role in the treatment of various diseases. However, the development of these drugs against neurological disorders where the drug target is located in the brain is challenging and requires a good understanding of the local drug concentration in the brain. In this original research, we investigated the systemic and local pharmacokinetics in the brain of healthy rats after either intravenous (IV) or intracerebroventricular (ICV) administration of EGFRvIII-T-Cell bispecific (TCB), a bispecific monoclonal antibody. We established an experimental protocol that allows serial sampling in serum, cerebrospinal fluid (CSF) and interstitial fluid (ISF) of the prefrontal cortex in freely moving rats. For detection of drug concentration in ISF, a push-pull microdialysis technique with large pore membranes was applied. Brain uptake into CSF and ISF was characterized and quantified with a reduced brain physiologically-based pharmacokinetic model. The model allowed us to interpret the pharmacokinetic processes of brain uptake after different routes of administration. The proposed model capturing the pharmacokinetics in serum, CSF and ISF of the prefrontal cortex suggests a barrier function between the CSF and ISF that impedes free antibody transfer. This finding suggests that ICV administration may not be better suited to reach higher local drug exposure as compared to IV administration. The model enabled us to quantify the relative contribution of the blood-brain barrier (BBB) and Blood-CSF-Barrier to the uptake into the interstitial fluid of the brain. In addition, we compared the brain uptake of three monoclonal antibodies after IV dosing. In summary, the presented approach can be applied to profile compounds based on their relative uptake in the brain and provides quantitative insights into which pathways are contributing to the net exposure in the brain.

## 1 Introduction

Over the last 20 years, more than 80 therapeutic monoclonal antibodies (mAbs) have been successfully developed in many therapeutic areas such as oncology, immunology, ophthalmology, genetic disease and neurological disorders (www.antibodysociety.org/antibody-therapeutics-product-data).

Those drugs have been established as a major class of therapeutics due to their distinct pharmacological characteristics. They bind with high specificity to its soluble or extracellular antigen and show a much lower risk of off-target toxicity as compared to small molecule drugs due to its high selectivity to the drug target (Wang et al., 2008; Castelli et al., 2019). The pharmacological response of monoclonal antibodies (mAbs) is driven by its potency and the drug concentration at the target site. Therefore, a good understanding of the pharmacokinetic processes is required to select a dose range that will result in relevant drug exposure at the target site (Eigenmann et al., 2021; Tang and Cao, 2021). Mabs distribute in the vascular and interstitial space and their extravasation into tissues is driven by convection and transcytosis (Ryman and Meibohm, 2017; Ovacik and Lin, 2018). Due to their high molecular weight, diffusion is not considered to be relevant for tissue uptake (Baxter et al., 1994). Instead, the lymphatic system plays a key role in removing cell protein and fluid from the tissue interstitial space and is critical for pressure regulation at a tissue and systemic level (Wiig and Swartz, 2012). After intravenous or subcutaneous administration, therapeutic monoclonal antibodies and Fc-fused protein exhibit prolonged systemic exposure via FcRn mediated recycling and transcytosis processes (Dirks and Meibohm, 2010). Mabs are cleared via a non-specific elimination pathway, which refers to target-independent, nonspecific cellular uptake followed by lysosomal degradation and subsequent removal from the circulation (Ovacik and Lin, 2018). In addition to this dose-linear clearance process, a non-linear clearance pathway has been widely observed for many therapeutic antibodies (Dirks and Meibohm, 2010). Here, the interaction with their target may result in receptor mediated endocytosis leading to fast removal of mAbs from circulation at non-saturating concentration. The rate and extent of this dose-dependent clearance depends on the antigen density, the affinity of the mAb to the antigen and the antigen turnover kinetics (Mager and Jusko, 2001).

One of the key hurdles for therapeutic antibodies developed to treat CNS disorders is to achieve relevant drug exposure at the site of action, which is the interstitial fluid of the brain region of interest. The brain uptake of antibody-based therapeutics is highly restricted (Strazielle and Ghersi-Egea, 2013) and a low brain to serum ratio in the range of 0.1%–1% has been reported (Wang et al., 2008; Le Prieult et al., 2021). Antibody-based therapeutics enter the brain via two barriers, namely the blood-brain-barrier (BBB), an endothelial barrier which is lining the cerebral microvessels and dispersed throughout the brain parenchyma, or the blood-CSF-barrier (BCSFB), an epithelial barrier in the choroid plexuses that is located within the ventricle (Le Prieult et al., 2021) and where the outer arachnoid epithelium plays a possible role in antibody entry from the plasma into the CSF. In addition, it is postulated that antibody based therapeutics can enter from the CSF compartment to the ISF of the brain. However, the relative contributions of both barriers to the net uptake into the brain remain unclear (Bloomingdale et al., 2021).

Measurement of relevant drug exposure in the brain is therefore critical, however challenging. Mostly this is done in preclinical studies by measurement of drug in homogenized brain tissue after whole body perfusion or by accounting for blood contamination (Abuqayyas and Balthasar, 2013). In the latter case the amount of drug in brain blood vessels is subtracted from the amount of drug measured in brain tissue. Both methods bear the risk of erroneous determination of brain concentrations in parenchyma. As an alternative approach, such as large pore membrane microdialysis (Chang et al., 2018; Janson et al., 2020) and open flow perfusion (Le Prieult et al., 2021) have been successfully applied to measure drug exposure in the interstitial fluid of different brain regions in nonclinical studies. In clinical studies, the PK is sampled from the cerebrospinal fluid (CSF) as a surrogate of brain exposure (Pestalozzi and Brignoli, 2000; Rubenstein et al., 2007; Stemmler et al., 2007), however this is not appropriately reflecting the drug concentration in the ISF of the brain (Pardridge, 2016). This discrepancy may be caused by the high perfusion of the choroid plexus (Kouhi et al., 2021)as well as the comparative leakiness of their capillary (Damkier et al., 2013; MacAulay et al., 2022).

Another question is related to the optimal route of administration to increase the brain exposure of antibody-based therapeutics. In the present work, we designed a mechanistic PK study that allows us to compare the uptake of a bispecific antibody as a tool compound after intracerebroventricular (ICV) or intravenous (IV) administration in rats using a push-pull microdialysis technique with large pore membranes. In this study, we tested EGFRvIII-TCB (Iurlaro et al., 2022), a T-cell bispecific antibody targeting the epidermal growth factor receptor variant III (EGFRvIII), which is currently under clinical development for the treatment of glioblastoma (https://clinicaltrials.gov/ct2/show/NCT05187624). The applied method allowed for serial sampling of drug concentration over 48 h in serum, CSF and ISF of brain parenchyma. In addition, we compared the brain uptake of EGFRvIII-TCB with two additional tool compounds (DP47-TCB and DP47-IgG) after IV administration. All three tested mAbs show no cross-reactivity with targets in the rat and therefore no target-binding is expected, so the disposition of the molecules can be compared based on their molecular properties. We selected the molecules since both, EGFRvIII-TCB and DP47-TCB have the same molecular format and molecular weight (MW = 195 kDa) but different antigen binding fragment (Fab) regions exhibiting different biophysical properties i.e. charge patches. DP47-IgG has a lower molecular weight (MW = 143 kDa) and contains the same two Fabs as DP47-TCB but lacks the CD3-binding Fab. For quantitative interpretation of the PK data and to provide insights into physiologically relevant processes in the brain uptake, we developed a reduced Physiologically-Based PK model (rPBPK) which was built and refined based on previous published models (Fronton et al., 2014; Chang et al., 2019; Bloomingdale et al., 2021). In conclusion, the proposed model allows us to quantify the relative contributions of the two barriers (BCSFB and the BBB) to the uptake of the tool compounds into the ISF of the brain and to estimate the transfer of the antibodies from CSF to ISFbrain.

## 2 Materials and methods

### 2.1 Test items

Three different monoclonal antibodies were used as tool compounds in this study: EGFRvIII-TCB (RO7428731, MW = 195 kDa), DP47-TCB (MW = 195 kDa) and DP47-IgG (MW = 143 kDa). EGFRvIII-TCB was designed in the 2 + 1 heterodimeric format that consists of two antigen binding fragments (Fab) specific for the EGFRvIII and one Fab recognizing the CD3Ɛ chain of the T-cell receptor as described (Bacac et al., 2016) and an Fc part devoid of FcɣR binding and CDC activity by introduction of P329G, L234A, L235A (PG-LALA) mutations (Schlothauer et al., 2016). DP47-TCB was designed in the analogous 2 + 1 heterodimeric format based on two Fabs containing a germline non-binding variable domain and one Fab containing a CD3Ɛ chain binding variable domain. DP47 IgG1, consisting of two germline non-binding variable domains and a Fc region bearing PG-LALA mutations. All binders used in these studies are not cross reactivity to targets in the rat.

### 2.2 *In vitro* recovery experiment

The relative recovery of antibodies using the push pull PP-PE 6/4 probes (CNS probes with a 4 mm polyethylene membrane, CRL Groningen, the Netherlands) was determined by *in vitro* recovery experiments. Probes were positioned in a beaker containing 1,000 ng/ml of antibody diluted in artificial CSF (aCSF −147 mM NaCl, 3.0 mM KCl, 1.2 mM CaCl2, 1.2 mM MgCl2) + 0.2% BSA. The beaker contents were continuously stirred and kept at a constant temperature of 37°C. The probes were positioned in the beakers and perfused with aCSF +0.2% BSA at a flow rate of 0.5 µl/min. Following pre-stabilisation, six microdialysis samples were collected in 30 min intervals. In addition, beaker content reference samples were collected at the start and the end of the experiment. Recovery was calculated as the average concentration in the microdialysate samples divided by the average concentration in the beaker samples. For EGFRvIII-TCB, an *in vitro* recovery rate of 14 % and 10% for DP47-IgG and for DP47-TCB were determined. The measured drug concentration in microdialysate was converted into the respective drug concentration in ISF of PFC by accounting for the respective *in vitro* recovery.

#### 2.3.1 Animals

A total of 25 adult male Wistar rats (Crl:WI CRL Sulzfeld, Germany; weight range 272–336 g) were used for the *in vivo* experiments. Experiments were conducted in strict accordance with the Guide for the Care and Use of Laboratory Animals (National Research Council 2011) and were in accordance with European Union directive 2010/63 and the Dutch law. The experiments were carried out under a license, issued by the national committee for licensing of animal experiments (Centrale Commissie Dierproeven) and were approved by the Animal Care and Use Committee (Instantie voor Dierenwelzijn) of CRL Groningen.

Following arrival, animals were housed in groups of up to five in polycarbonate cages (65 × 33 × 20 cm) with wire mesh top in a temperature (22 ± 2°C) and humidity (55% ± 15%) controlled environment on a 12 h light cycle (07.00–19.00 h). After surgery, animals were housed individually in polypropylene cages (30 × 30 × 40 cm). Standard diet (SDS Diets, RM1 PL) and domestic quality mains water were available *ad libitum*.

#### 2.3.2 Surgery, microdialysis probe implantation and cannulation

Animals were anesthetized using isoflurane (2.5%–3% and 800 ml/min O2). Before surgery, flunixin (MSD, 1 mg/kg, subcutaneous) was administered once for analgesia during surgery and the post-surgical recovery period. A mixture of bupivacaine and epinephrine was applied to the incision site and to the periost of the skull for local analgesia.

Each animal was placed in a stereotaxic frame (Kopf Instruments, United States) and PP-PE 6/4 microdialysis probes (CRL Groningen, the Netherlands) were positioned in the left prefrontal cortex (PFC; coordinates for the tip of the probe: AP = +3.4 mm from bregma, lateral +0.8 mm from midline and ventral −5.0 mm from dura). In addition, an injection guide suited for compound administration (CRL Groningen, the Netherlands), was positioned in the right lateral ventricle (LV; coordinates for the tip of the probe: AP = −0.8 mm from bregma, lateral +1.8 mm from midline and ventral −4.0 mm from dura). All coordinates were based on (George Paxinos, 2006).

In the same surgical procedure an indwelling cannula was placed in the jugular vein (JV; 42 mm CRL Groningen, the Netherlands) to allow for blood sample collection. A stainless steel cannula was placed in the cisterna magna (CM; CRL Groningen, the Netherlands) to allow for CSF sample collection. The probe, injector guide, and cannula were fixed in position and attached to the skull with stainless steel screws and dental cement.

#### 2.3.3. Compound administration and sample collection

After a day of recovery, the push pull probes were connected to a microperfusion pump (Harvard Apparatus, United States) with PEEK tubing (Western Analytical Products Inc. United States; PK005-020). The probes were perfused with a CSF +0.2% BSA at a flow rate of 0.5 µl/min. Following 2 h of pre-stabilization, microdialysate sample collection was initiated. At t = 0 min, antibody in physiological buffer formulation (20 mM His/His-HCl, 240 mM Sucrose, 10 mM Methionine, 0.05% PS, pH = 5.50) was administered intracerebroventricular via the injection guide (1 or 3 mg/kg, both at a flow rate of 3 µl/min formulated at concentration of 20 mg/ml) or intravenously (15 mg/kg at 5 ml/kg *via* the tail vein). Microdialysates were collected in 30-min intervals during the first 8 h post-dose. From 8 h post-dose until 48 h post-dose samples were collected in 4-h intervals. Blood samples and CSF samples were collected at t = −0.5, 0.5, 1, 3, 4.5, 6, 12, 24, and 48 h post-dose via the respective cannulae positioned in the jugular vein and cisterna magna. Blood samples were allowed to clot and centrifuged to obtain serum.

One day of recovery has been established at CRL facilities to be sufficient for the BBB to close and any occurring perioperative effects to dissipate. In house measurements quantifying stress (e.g., HA and 5-HT) and inflammation markers (e.g., adenosine and IL-1beta) were at baseline after 1 day of recovery. Push pull microdialysis procedures are performed using a gravity pull and concomitant validation of the flow by weighting each sample, rather than the active pull using a perfusion pump. Both in house historic and published data (de Lange et al., 2000) support the continuous use of the microdialysis probe for up to 48 h without gliosis.

The experiments were performed in freely moving awake animals which were continuously monitored. No behavioral change was observed during or immediately following the administrations by infusion suggesting there was no marked change in pressure on the ventricle. In addition, prior to dose administration a basal CSF sample was collected which will have temporarily caused a minor decrease in total CSF volume, and thus reducing potential overall relative volumetric concerns. Similar administrations in the lateral ventricle have shown no effects on pressure or change to ventricular size as followed by MRI (Murtha et al., 2014).

### 2.4 ELISA to quantify drug concentration in serum, CSF and ISF brain

Bioanalytical assays were developed for measurement of the three tested drugs (EGFRvIII-TCB, DP47-TCB or DP47-IgG) in rat serum samples using an enzyme-linked immunosorbent assay (ELISA). Capture of EGFRvIII-TCB was done with biotinylated EGFRvIII antigen, while DP47-TCB or DP47-IgG was captured by an anti-DP47 antibody. The bound drug was detected using digoxigenin-labeled anti-PG antibody (Wessels et al., 2017) followed by addition of an anti-Dig-POD secondary detection antibody. Signals were generated by addition of peroxidase substrate. For the EGFRvIII-TCB assay, calibration range was 2.35 ng/ml to 150 ng/ml with the lower limit of quantification (LLOQ) being 2.5 ng/ml. For the DP47-TCB (DP47-IgG) assay, the calibration range was 1.4–90 ng/ml with LLOQ of 1.4 ng/ml.

### 2.5 Data analysis

#### 2.5.1 Non-compartmental analysis

Noncompartmental analysis (NCA) was performed in MATLAB Simbiology (version R2021b) using the linear trapezoidal rule for interpolation. Each rat was treated as an individual subject and NCA parameters have been reported as mean ± standard deviation. Areas under the concentration versus time curves (AUC0→t-last) in serum, ISFbrain, and CSF were calculated for the tested antibodies. The maximal drug concentration in serum (Cmax) and time to maximal concentration (Tmax) were derived.

#### 2.5.2 Reduced brain PBPK model structure

A reduced brain PBPK (rPBPK) model was built to describe the uptake of mAbs into CSF and ISFbrain after ICV and IV dosing. The final model structure is shown in Figure 3. It was inspired by previously published brain PBPK models (Chang et al., 2019; Bloomingdale et al., 2021). We further reduced the model as proposed by Fronton and colleagues (Fronton et al., 2014) since we did not have the granularity in our data to separate between transcytosis and paracellular transport. Since the data did only reflect net exposure levels, the cellular-endocytosis model for mAb degradation in the brain was not included.

The following components of the structural model were included: 1) the model accounted for the physiological compartments such as brain vascular and interstitial space of the brain parenchyma, the CSF compartment consisting of lateral ventricles (LV), third and fourth ventricles (TFV), cisterna magna (CM), and subarachnoid space (SAS), serum, lymph, and a lumped rest-of-body compartment. 2) The brain compartment was described with volumes of CSF and ISFbrain compartments. 3) Brain uptake was described by convection through large pores and transcytosis. These processes are governed by the physiological flows in the brain. A reflection coefficient (1- σ) with σ ∈ [0,1] is also added to account for permeability limitations for molecules across the brain barriers. These reflection coefficients are drug-related parameters and vary between different mAbs. 4) The model further assumes a barrier function between the CSF and ISFbrain, which is captured with another reflection coefficient limiting the flow of antibody from CSF to the ISFbrain. The final structural model is presented in Figure 3.

The model structure has the following assumptions:

1) The brain is described by three main components: brain vasculature, CSF compartment, and the ISFbrain in the brain parenchyma. The CSF compartment is subdivided into the lateral ventricles (LV), third and fourth ventricles (TFV), cisterna magna (CM), and subarachnoid space (SAS). This was done to account for ICV dosing that is administered into the LV, while CSF sampling took place in CM. The respective physiological parameters such as CSF flows and compartment volumes were taken from (Chang et al., 2019). Except for the brain, serum, and lymphatic system, all other tissues were lumped together as a rest-of-body compartment. This compartment was subdivided into a tissue vascular compartment, tissue endosomal compartment, and tissue interstitial compartment (Bloomingdale et al., 2021). The model assumes that the antibodies extravasate from the brain vasculature into the CSF at the level of the choroid plexus in the ventricles, which forms the BCSFB, and into the ISF at the level of the cerebral vascular endothelium, which forms the BBB. Antibodies within the CSF follow the CSF flow towards the subarachnoid space and then through the ependymal cell layer towards the interstitial space of the brain parenchyma. The exact mechanisms behind the flow of CSF towards the ISF and the clearance from those compartments are highly debated. One widely discussed hypothesis is that of the glymphatic system, which postulates the existence of a convective flow of CSF into the brain through the perivascular space of penetrating arteries. Active transport enables CSF contents to reach the brain extracellular matrix and mix with ISF. In turn, ISF can be reabsorbed through a perivenous pathway and cleared (Iliff et al., 2012; Albargothy et al., 2018; Aldea et al., 2019; Mestre et al., 2020; Mestre et al., 2020). Although recently emerging evidence supports the existence of such a perivascular fluid system, there are reasons to consider that diffusion rather than convection plays a crucial role here (Abbott et al., 2018). Based on these hypotheses and lack of more detailed information, the current model assumes a unilateral flow from CSF to ISF at the level of SAS and antibody clearance from both SAS and ISF towards the lymphatics, which is in line with previously published models. In addition, the serum volume was derived from the body weight-corrected blood volume in rats (Mandikian et al., 2018), assuming a mean body weight of 310 g and a hematocrit of 0.45. All physiological parameters values are displayed in Table 1.
2) Since there is only negligible elimination through lysosomal degradation expected in the brain (Eigenmann et al., 2017b), there was no elimination pathway of the brain included in the proposed model structure. This was supported by a pathway analysis of the original brain mPBPK model (Bloomingdale et al., 2021), which confirms the limited contribution of elimination in the brain to overall systemic clearance (Supplementary Figure S1).
3) The uptake into the CSF and ISFbrain of the brain parenchyma were described as net uptake, and no distinction between transcellular and paracellular transport was made. The net brain uptake was described as a lumped or hybrid reflection coefficient (σuptake) across either the BBB or BCSFB.
4) A reflection coefficient was included to restrict the flow of antibodies between the CSF and ISFbrain (σCSF_ISF).

**TABLE 1:** Model Parameters and their corresponding values, units and physiological meaning.

Parameter	Value (rat)^a^	Units	Physiological meaning
BrainISF	4.1e-4	L	Volume of brain interstitial fluid
BrainVasc	5.02e-5	L	Plasma volume in brain vasculature
CM	1.7e-5	L	Volume of cisterna magna
FcRnSS	49,800	nM/L	Steady state concentration of FcRn in endosomes
FR	0.715	-	Fraction of FcRn-bound antibody in endosome that is recycled back to the plasma space
kCLupT	0.55	1/h	Uptake rate of antibody into the tissue endosomal space
Koff, FcRn	144	1/h	Dissociation rate of FcRn-binding
kon,FcRn	0.8	nM/h	Association rate of FcRn-binding
Lb	1.62e-4	L/h	Brain lymph flow
Lt	0.0058	L/h	Tissue lymph flow
LV	5e-5	L	Combined volume of both lateral ventricles
Lymph	0.0011	L	Lymph volume
Plasma	0.0067	L	Plasma volume
Qb	0.0653	L/h	Brain blood flow
QBCSF	1.32e-4	L/h	CSF circulation/production flow
QBISF	3.0e-5	L/h	ISF circulation/production flow
Qt	2.88	L/h	Tissue blood flow
SAS	1.8e-4	L	Volume of subarachnoid space
TFV	5e-5	L	Volume of third and fourth ventricle
TissueEndo	0.0013	L	Volume of tissue endosomal space
TissueISF	0.0483	L	Volume of tissue interstitial fluid
TissueVasc	0.0079	L	Plasma volume in tissue vasculature
σBISF	0.2	-	reflection coefficient on outflow from brain ISFbrain
σCSF	0.2	-	Reflection coefficient on outflow from SAS
σTL	0.2	-	Lymphatic reflection coefficient from tissue ISF
σTV	0.9212	-	Reflection coefficient for entering tissue ISF from vasculature
σuptake,BBB	Drug dependent, estimates see Table 3	-	Reflection coefficient for entering brain ISF from vasculature through BBB
σuptake,BCSFB		-	Reflection coefficient for entering CSF from vasculature through BCSFB
σCSF_ISF		-	Reflection coefficient for transfer through barrier between CSF and ISF
kdeg		1/h	Endosomal degradation rate

a All non-estimated parameter values were taken as reported by (Chang et al., 2019) with the exception of plasma volume (Plasma) which was taken as reported by (Mandikian et al., 2018).

In line with existing knowledge on CSF flow physiology (Iliff et al., 2012; Mestre et al., 2020) and as captured by Chang et al. (2019) in the full PBPK model, antibody enters the ISFbrain from the CSF at the level of the subarachnoid space (SAS) and cycles back to the CSF at the level of the lateral ventricle (LV) and third and fourth (TFV) ventricles. This flow from ISF to CSF is caused by the formation of ISFbrain at the BBB which contributes for a small part to CSF formation and gives rise to a fluid flow driven by the ISFbrain production rate (QISF) (Abbott, 2004). The net uptake via convection is captured with

QISF∗(1-σCSF_ISF)

Where σCSF_ISF governs the fraction of drug that is prevented from crossing the CSF-ISF barrier.

#### 2.5.3 Parameter estimation

The newly developed reduced PBPK model consists of 13 ODEs and 32 parameters. Of those parameters, 28 were fixed to reported physiological values (Shah and Betts, 2012; Mandikian et al., 2018; Bloomingdale et al., 2021) and four drug-related parameters, namely logit transformed reflection coefficients and the endosomal degradation rate kdeg were estimated. Parameters are summarized in Table 1. The model was developed in MATLAB Simbiology version R2021b. The ODE solver used was ode15s. The local solver lsqnonlin was used for parameter estimation. Local sensitivity analysis was performed with the build-in tool in Simbiology. Simulations and pathway analyses were performed in Berkeley Madonna (version 8.13.18) using the Rosenbrock solver.

The rat PK data from EGFRvIII-TCB, DP47-IgG, and DP47-TCB were modeled separately and using a two-staged approach. For EGFRvIII-TCB, a naive-pooled approach was used to fit the model to the rat data (*n* = 15). First, the model was fit to serum PK data after IV dosing in order to estimate the endosomal degradation rate (kdeg). A proportional error model was used. The obtained parameter value was then fixed and three parameters related to brain uptake (σuptake,BBB, σuptake,BCSFB, and σCSF_ISF) were estimated by fitting the model simultaneously to CSF, and ISFbrain data sampled from the three dosing groups of EGFRvIII-TCB (15 mg/kg IV, 3 mg/kg ICV, and 1 mg/kg ICV). A constant error model was assumed.

A similar modeling strategy was applied, independently, for DP47-IgG (rats *n* = 5) and DP47-TCB (rats *n* = 5). First, the model was fit to serum PK data in order to estimate the systemic elimination kdeg. A proportional error model was used. In the second step, only two drug-related parameters (σuptake,BBB, σuptake,BCSFB) were estimated while the third parameter (σCSF_ISF) was fixed to the value obtained from EGFRvIII-TCB. The model was fitted simultaneously to CSF, and ISFbrain data sampled after 15 mg/kg IV dosing. A constant error model was assumed. In order to provide a quantitative assessment of model performance, we calculated the prediction error (PE%) using the equation

$$\text{PE}\left(\text{\%}\right)=\frac{AUC_{predicted}-AUC_{observed}}{AUC_{observed}}\text{×}100\text{\%}$$

As described in (Chang et al., 2019), where AUC denotes the area under the curve for the given observation period.

#### 2.5.4 Pathway analysis

Pathway analysis for elimination was performed to quantify the contribution of the local drug clearance in the brain to total body clearance of the EGFRvIII-TCB using the model presented in (Bloomingdale et al., 2021). This was done by comparing the total amount eliminated with the rate kdeg in the endosomes in the brain (Eq. 1) and the other tissues (Eq. 2):

$$\frac{d\;Cleared_{BRAIN}}{dt}=k_{deg}\times \left(Drug_{endosome,BBB}\times V_{endosome,BBB}+Drug_{endosome,BCSFB}\times V_{endosome,BCSFB}\right)$$ (1)

$$\frac{d\;Cleared_{TISSUE}}{dt}=k_{deg}\times Drug_{endosome,tissue}\times V_{endosome,tissue}$$ (2)

With kdeg the endosomal degradation rate of unbound antibody, Drugendosome the antibody concentration in the endosomes that make up either the BBB, BCSFB, or all other tissues. Vendosome is the total endosomal volume in either the BBB, BCSFB, or all other tissues.

The contribution of the brain to total body clearance of EGFRvIII-TCB over time was then calculated up to 48 h as:

$$Clearance_{BRAIN\%}=\frac{Cleared_{BRAIN}}{Cleared_{TISSUE}\;+\;Cleared_{BRAIN}}\times 100\%$$ (3)

Pathway analysis for relative contribution was also used to quantify the relative contribution of EGFRvIII-TCB entering the ISFbrain from either serum or CSF. This was mainly driven by the respective reflection coefficients (Eqs 4, 5).

$$\frac{d\;Serum\to ISF}{dt}=\left(1-\sigma_{uptake,BBB}\right)\times Q_{ISF}\times Drug_{Serum}$$ (4)

$$\frac{d\;CSF\to ISF}{dt}=\left(1-\sigma_{CSF\;ISF}\right)\times Q_{ISF}\times Drug_{SAS}$$ (5)

Where DrugSerum and DrugSAS are the drug concentrations in serum and the subarachnoid space, respectively. Eqs 4, 5 were numerically simulated over time and their AUC was calculated using the linear trapezoidal rule. Their relative contributions were calculated as $\frac{AUC_{\left\{Serum\to ISF\right\}}}{AUC_{\left\{CSF\to ISF\right\}}\;+\;AUC_{\left\{Serum\to ISF\right\}}}$ for the serum pathway and $\frac{AUC_{\left\{CSF\to ISF\right\}}}{AUC_{\left\{CSF\to ISF\right\}}\;+\;AUC_{\left\{Serum\to ISF\right\}}}$ for the CSF pathway.

These fractions were then multiplied with the relative exposure in ISF compared to serum, which was calculated as:

$$ISF/serum\%=\frac{AUC_{ISF}}{AUC_{Serum}\;+\;AUC_{ISF}}\times 100\%$$ (6)

### 2.6 In silico analysis of 3D charge distribution

The amino acid sequences of the variable domain VH of the EGFRvIII binder and the DP47 mAb were used to create a homology model using the MoFvAb software version 10 (Bujotzek et al., 2015). By using an in silico calculation method starting with the homology model, followed by pH- protonation of acidic and basic side-chains, we calculated the 3D charge distribution using the software CHARMM and Delphi as implemented in the software suite Discovery Studio (vendor: Dassault Systems).

## 3 Results

### 3.1 Brain uptake after ICV versus IV dosing

**FIGURE 1:**
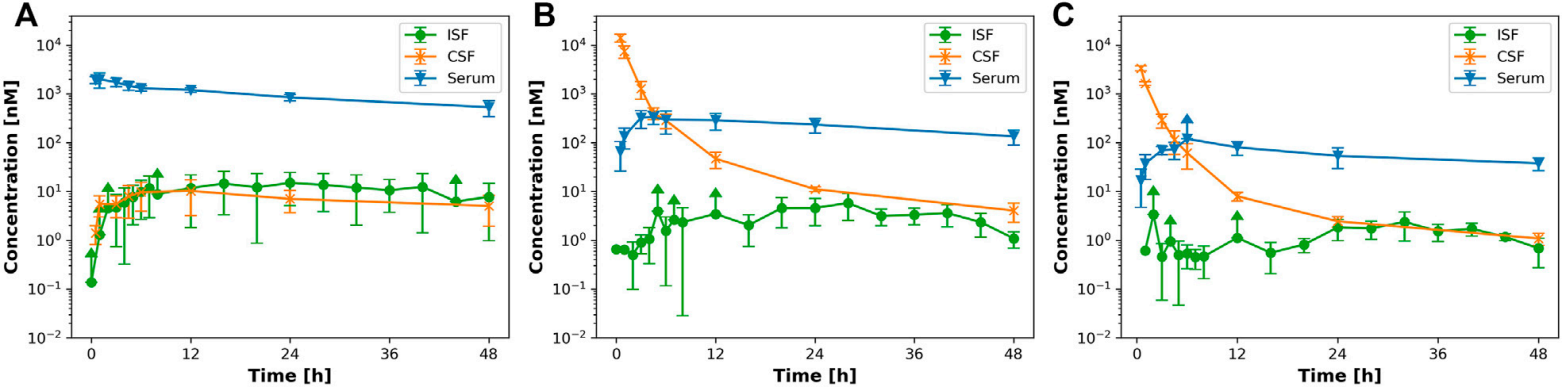
IV versus ICV dosing for EGFRvIII-TCB. Figure 1 shows the PK profile after **(A)** 15 mg/kg iv; **(B)** 3 mg/kg ICV and **(C)** 1 mg/kg icv of EGFRvIII-TCB in serum (blue), ISFbrain (green) and CSF (orange).

In order to investigate brain uptake after IV or ICV administration, we conducted a PK study in rats and collected the PK profiles in serum, CSF, ISFbrain of prefrontal cortex (Figure 1). After IV dosing (15 mg/kg, Figure 1A), the highest concentration was achieved in serum, whereas after ICV dosing, the highest concentration was observed in CSF. For quantitative comparison of the relative local exposures, we computed the area under the concentration over 48 h (AUC0-48 h) in addition to the maximum concentration (Cmax). The results are displayed in Table 2. The relative exposure in ISFbrain and CSF versus serum were comparable after IV dosing. However, after ICV dosing, the exposure in ISFbrain amounted to only between 0.7% and 1.2% of CSF.

**TABLE 2:** Results obtained by non-compartmental analysis (NCA) PK analysis.

Compound	Dose	Cmax (±SD)	Tmax	Serum AUC0-48 (±SD)	CSF	ISF	AUC ratio CSF/serum	AUC ratio ISF/serum	AUC ratio ISF/CSF
					AUC0-48 (±SD)	AUC0-48 (±SD)			
	mg/kg	nM	h	nM×*h*	nM×*h*	nM×*h*	%	%	%
EGFRvIII-TCB	15, IV	2,237 (350)	0.5–3	46,618 (4,065)	345 (143)	555 (384)	0.74	1.19	161
	3, ICV	358 (126)	3–6	10,862 (3,618)	22,495 (7,317)	150 (67)	207	1.38	0.67
	1, ICV	126 (126)	3–12	2,794 (1,318)	5,402 (676)	64 (31)	193	2.29	1.18
DP47-IgG	15, IV	3,358 (757)	1–2	85,906 (10,736)	674 (747)	465 (228)	0.78	0.54	70
DP47-TCB	15, IV	2,743 (347)	1–2	77,149 (6,727)	676 (482)	251 (138)	0.88	0.33	37

**FIGURE 2:**
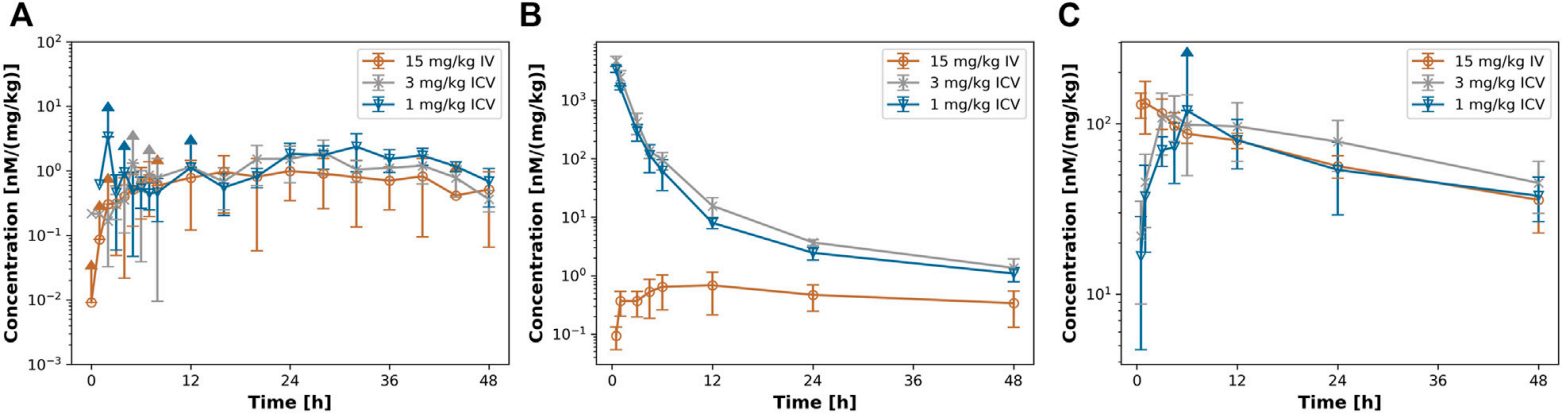
dose-normalized PK profiles after iv or icv dosing. Figure 2 shows the dose normalized PK profile in **(A)** ISFbrain. **(B)** CSF and **(C)** serum after 15 mg/kg iv (orange) 3 mg/kg ICV (grey) and 1 mg/kg icv (blue) of EGFRvIII-TCB.

Figure 2 shows the dose-normalized PK profiles in serum (Figure 2A), CSF (Figure 2B), and ISFbrain (Figure 2C). Interestingly, in serum the dose-normalized PK profiles superimpose for both routes of administration after the maximum concentration has been reached with ICV dosing. In addition, the dose-normalized PK in ISFbrain of all three groups overlapped. The dose-normalized PK profile in CSF is the same after 1 mg/kg and 3 mg/kg suggesting linear processes. As expected, the dose-normalized PK profile in CSF after ICV administration is significantly higher than after IV injection.

Non-compartmental analysis showed that the maximal serum concentrations (Cmax) were similar for EGFRvIII-TCB (2,237 ± 350 nM), DP47-IgG (3,358 ± 757 nM), and DP47-TCB (2,743 ± 347 nM). However, total drug exposure reported as AUC0-48h of EGFRvIII-TCB was 40%–46% lower than those of DP47-TCB and DP47-IgG, respectively. The exposure in ISFbrain versus serum was overall higher for EGFRvIII-TCB (0.41–1.71%) compared to DP47-IgG (0.19%–0.87%) and DP47-TCB (0.16%–0.64%). The NCA results are summarized in Table 2.

### 3.2 Reduced PBPK model predicts PK profiles after ICV and IV administration

Next, we compared how well the recently proposed minimal PBPK (mPBPK) model (Bloomingdale et al., 2021) predicts the observed PK profiles in the different tissue compartments after IV and ICV dosing. The model adequately captures the PK of EGFRvIII-TCB after IV administration (Supplementary Figure S2A). However, the model failed to capture the ISFbrain PK after ICV dosing and overpredicted the exposure by several orders of magnitude (Supplementary Figures S2B,C; Supplementary Table S1).

**FIGURE 3:**
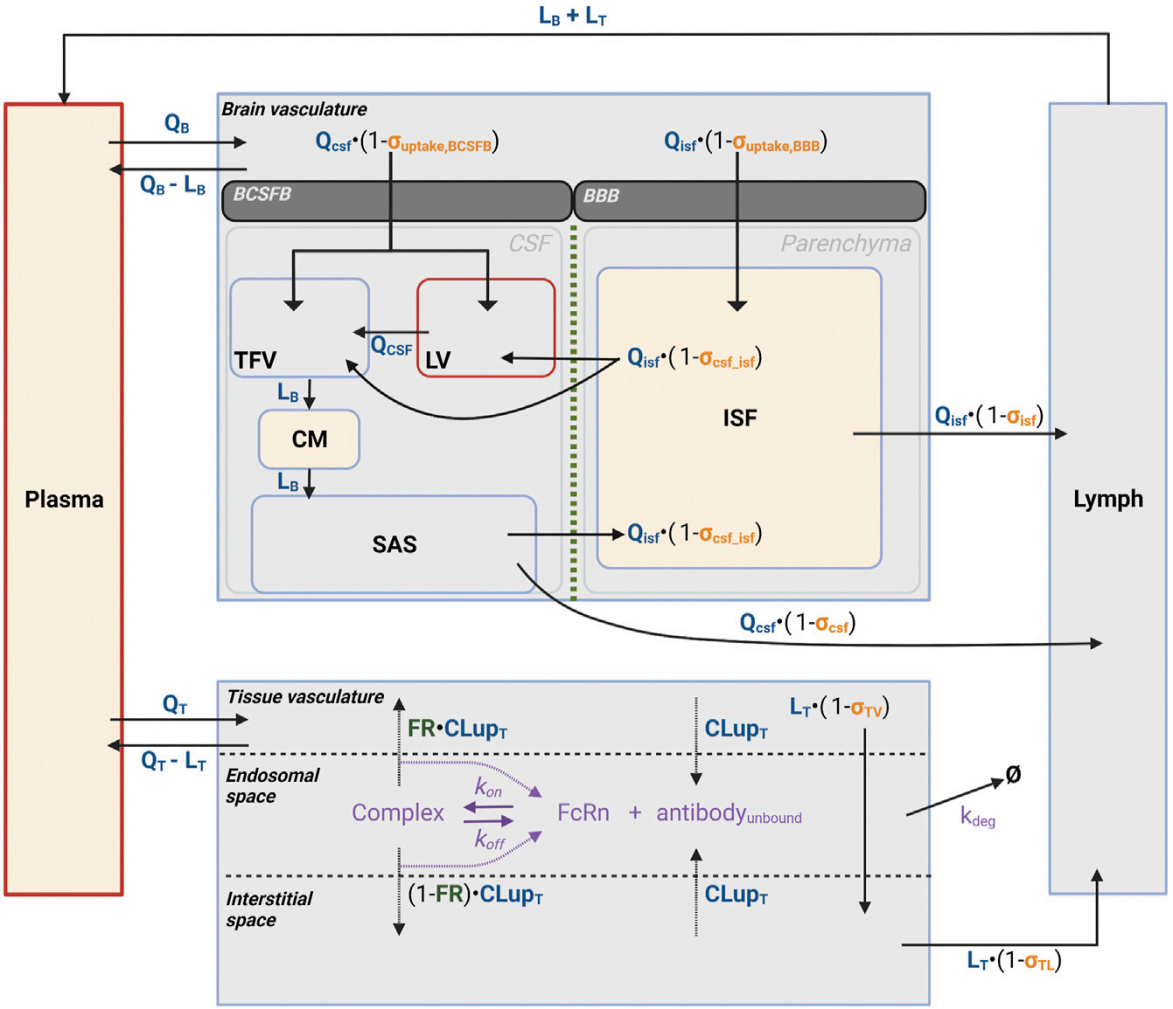
Reduced brain PBPK model structure. Figure 3 depicts the schematic representation of the reduced PBPK model structure to characterize antibody disposition in brain vasculature towards CSF and ISF. Antibodies flow *via* plasma to CSF, ISFbrain or interstitial space in the brain and leave via lymphatic flow. Disposition and clearance from tissue has been described as introduced by Bloomingdale et al. (2021). In tissue, antibodies enter the interstitial space *via* paracellular uptake or transcellular via endosomal uptake. In the endosome, antibody binds FcRn to form an antibody-FcRn complex, which either is taken up into the tissue interstitial space or recycled back to the tissue vasculature. Antibody can enter the brain through the BCSFB to enter the CSF in the lateral (LV) or third and fourth (TFV) ventricles, or through the BBB to enter the ISF in the brain parenchyma. Antibody can travel between the CSF and ISF through the CSF-ISF barrier. Yellow shaded: measurement sites; Red border: injection sites.

**FIGURE 4:**
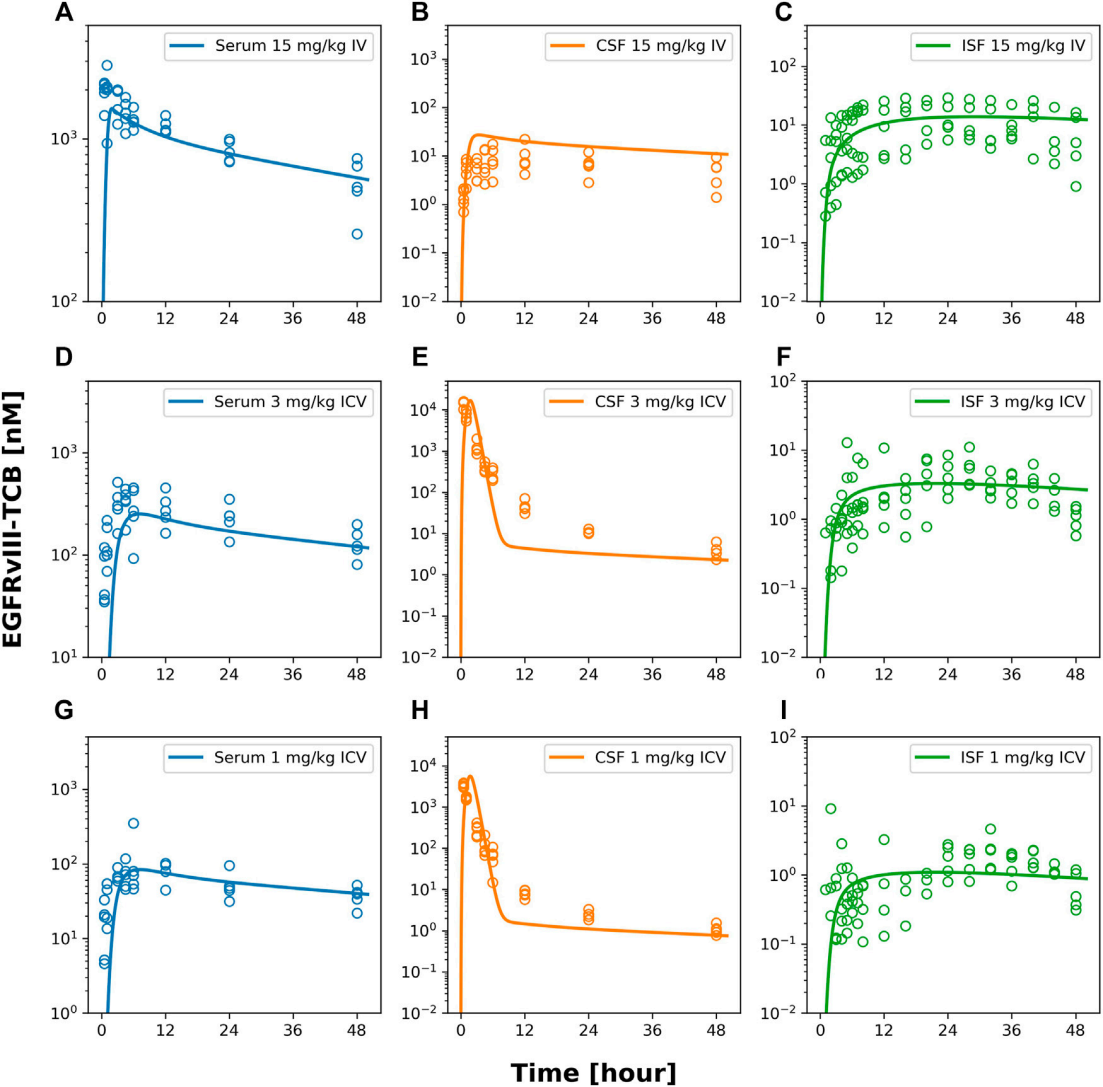
model fits of EGFRvIII-TCB. Figure 4 shows model predicted (solid lines) and individually observed PK data (symbols) after 15 mg/kg iv **(A–C)**, 3 mg/kg icv **(D–F)** or 1 mg/kg icv **(G–I)** of EGFRvIII-TCB in serum **(A,D,G)**, in CSF (orange, **(B,E,F)**) and ISFbrain (green, **(C,F,I)**).

Based on these insights, we developed and evaluated our proposed reduced brain PBPK model (Figure 3) and assessed how well the model predicted the observed data (Figure 4). We compared the performance of both models using their prediction errors summarized in Supplementary Table S1. The results suggest that the proposed rPBPK model improves the prediction of ISFbrain PK after ICV dosing.

Furthermore, the model described the PK of EGFRvIII-TCB in serum, CSF, and ISFbrain after IV administration of 15 mg/kg (Figures 4A–C), and after ICV administration of EGFRvIII-TCB at doses 3 mg/kg (Figures 4D–F) and 1 mg/kg (Figures 4G–I) by simultaneously fitting. The predictions and the observed data were in good agreement, with an average absolute prediction error of 42% (Supplementary Table S1). Model parameter estimates are displayed in Table 3. The parameter denoting the reflection coefficient on the net transfer from brain vasculature to ISFbrain (σuptake, BBB) was estimated to be 0.9853. The parameter denoting the reflection coefficient on the net transfer from brain vasculature to CSF (σuptake, BCSFB) was estimated to be 0.9767. Finally, the parameter denoting the reflection coefficient on the transfer between CSF and ISFbrain (σCSF_ISF) was estimated to be 0.9994. All reflection coefficients were estimated with good precision (%CV < 30%). Since the reflection coefficients were logit-transformed for estimation, the parameter values are constrained between 0 and 1. Model observed-versus-predicted plots are found in Supplementary Figure S3. The precision of parameter estimation for the endosomal degradation rates (kdeg) was low for all three molecules (reflected by %CV > 50%).

**TABLE 3:** Results of model parameter estimation.

Drug	Parameter	Estimate	CV%
EGFRvIII-TCB	kdeg	82.4	57.5
	σuptake,BBB	0.9853^a^	23.99
	σuptake,BCSFB	0.9767^a^	29.11
	σCSF_ISF	0.9994^a^	7.044
DP47-IgG	kdeg	27.3	152
	σuptake,BBB	0.9919^a^	0.0657
	σBCSFB	0.9865^a^	0.2027
DP47-TCB	kdeg	7.76	410
	σuptake,BBB	0.9946^a^	0.0304
	σuptake,BCSFB	0.9855^a^	0.1013

a Parameters are logit-transformed, constraining their values between (0,1).

### 3.3 Relative contribution of BBB and BCSFB barriers for brain uptake

**FIGURE 5:**
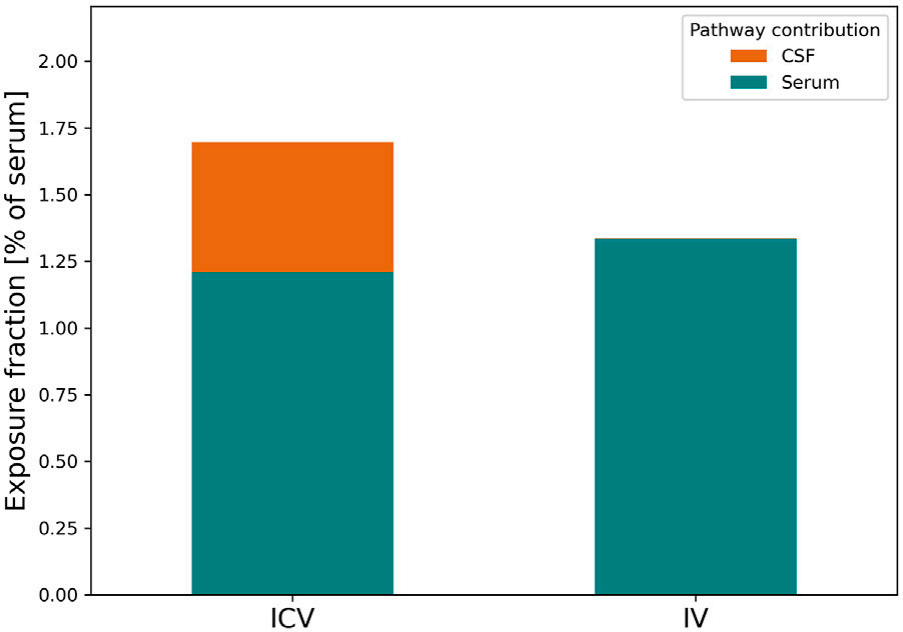
Relative contribution of CSF and plasma pathways to brain ISF exposure for EGFRvIII-TCB upon IV or ICV dosing. Figure 5 depicts the relative contribution of antibodies originating from CSF (orange) and serum (teal) to the total ISFbrain exposure after ICV (left) or IV (right) dosing. The relative contributions are expressed as the fraction of exposure in ISFbrain relative to serum.

Based on the model prediction, we investigated the relative contribution of the BBB or BCSFB to the brain uptake after IV or ICV dosing. First, we calculated the ratio of antibody exposure in ISFbrain versus serum (as shown in Eq. 6). The model predicts that over a 48 h period, the ISFbrain is exposed to 1.4% of total serum exposure upon IV dosing and 1.6% upon ICV dosing. We integrated over time the drug concentration reaching the ISFbrain from either serum and the subarachnoid space in order to quantify the percentage of drug entering from the brain vasculature via the BBB (Eq. 4) and *via* the CSF-ISF barrier (Eq. 5), respectively. The results from this pathway analysis are summarized in Figure 5. After IV administration, the model predicts that the main entry into the brain ISFbrain is via the BBB (99.9%). After ICV administration, 77.6% of antibodies reached the ISFbrain via serum whereas the remaining 22.4% reached the ISFbrain directly from CSF through the CSF-ISFbrain barrier.

### 3.4. Head to head comparison of EGFRvIII-TCB, DP47-TCB and DP47-IgG

**FIGURE 6:**
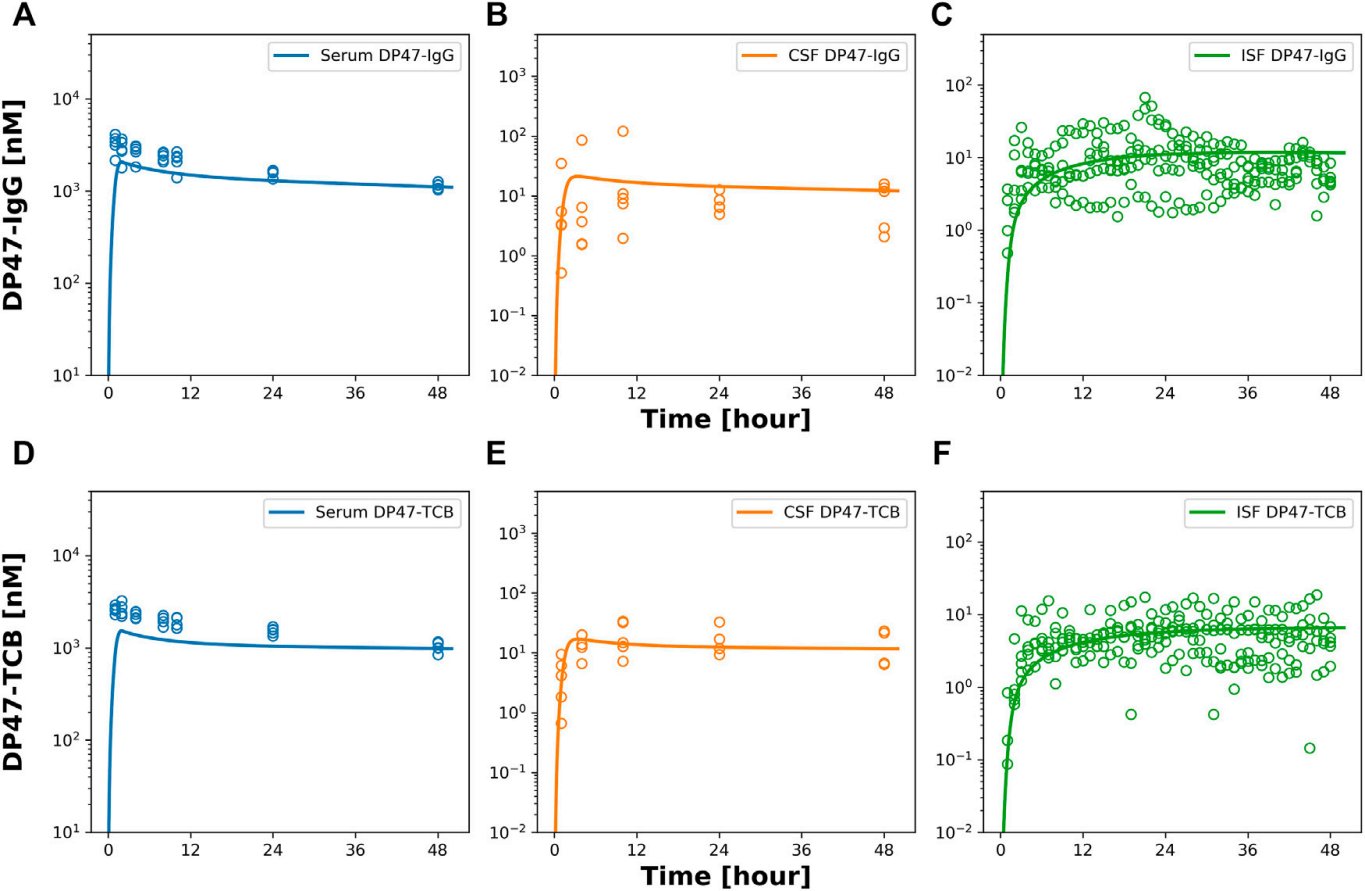
Observed and model predicted PK in serum, CSF and ISF after 15 mg/kg iv dosing of DP47-IgG and DP47-TCB. Figure 6 shows model predicted (solid lines) and individually observed PK data (symbols) after 15 mg/kg iv dosing of DP47-IgG **(A–C)** and after 15 mg/kg iv dosing of DP47-TCB **(D–F)** in serum (blue, **(A,D)**) in CSF (orange, **(B,E)**) and ISFbrain (green, **(C,F)**).

Next, we conducted a PK study with two additional tool compounds, DP47-TCB and DP47-IgG, and compared their relative brain uptake to EGFRvIII-TCB. The three compounds differ in their molecular properties namely format and therefore molecular weight as well as variable fragment (Fv) amino acid sequence and therefore biophysical properties like surface charge. While EGFRvIII-TCB and DP47-TCB have the same molecular format (2 + 1) and molecular weight (MW = 195 kDa), EGFRvIII-TCB has a positive charge patch in the Fab regions specific for the EGFRvIII (Supplementary Figure S4) which is not found on the DP47 Fab. DP47-IgG (1 + 1 format) has the same Fab region as DP47-TCB but is lacking the additional Fab with CD3Ɛ specificity and therefore has a lower molecular weight (MW = 143 kDa). PK profiles were collected in serum, CSF and ISFbrain after IV dosing of 15 mg/kg. Figure 6 shows the observed data (symbols) overlaid with the predicted PK profiles (solid lines). The reduced PBPK model was fitted simultaneously to the PK profiles in CSF and ISFbrain. This was done independently for both drugs. Most parameters were fixed to the values reported by (Bloomingdale et al., 2021), with σCSF_ISF fixed to the value estimated from EGFRvIII-TCB. The parameters σuptake, BBB and σuptake,BCSFB and kdeg, were estimated for both drugs independently. The final model was able to characterize the PK of DP47-IgG (Figures 6A–C) and DP47-TCB (Figures 6D–F) in serum, CSF, and ISFbrain after IV administration of a 15 mg/kg dose. The corresponding reflection coefficients σuptake, BBB and σuptake,BCSFB were estimated with good precision (%CV <20%) for all three drugs (Table 3). The lowest reflection coefficient for the BBB was predicted for EGFRvIII-TCB with (σuptake,BBB = 0.9853), followed by DP47-IgG (σuptake,BBB = 0.9919) and DP47-TCB (σuptake,BBB = 0.9946) suggesting that EGFRvIII-TCB has the highest relative uptake into the brain via the BBB. This result is in line with an additional quantification of the relative uptake that is based on the relative exposure ratios of CSF or ISFbrain over serum, as determined with non-compartmental analysis (Table 2). These ratios are 1.19%, 0.54%, and 0.33%, respectively, for EGFRvIII-TCB, DP47-IgG, and DP47-TCB.

## 4 Discussion

In the present work, we conducted a mechanistic PK study to assess and quantify the brain uptake of EGFRvIII-TCB after IV and ICV dosing in rats. In addition, we compared the brain uptake after IV dosing of EGFRvIII-TCB with two other tool compounds. In order to quantify the local drug exposure in the brain, we collected CSF by sampling from the cisterna magna and applied a push-and-pull microdialysis system using microdialysis probes with high molecular weight cutoff membranes that allow recovery of antibodies and proteins from the interstitial fluid (Rosdahl et al., 2000; Clough, 2005; Jadhav et al., 2016; Jadhav et al., 2017) of the prefrontal cortex. The key insights generated with the reduced brain PBPK model were related to the quantification of mAb uptake *via* BBB or BCSFB as well as their respective relative contributions to the uptake into the ISFbrain. The proposed model has a reduced complexity and may facilitate the quantitative interpretation of drug uptake through the BBB and BCSF barriers. This will in turn allow for easier cross-compound comparison, as we illustrated with the three compounds investigated.

While microdialysis is a well-established technique for small molecules, its use to measure antibody-based therapeutics in the brain has been quite limited until recently (Jadhav et al., 2016; Kouhi et al., 2021). Although the push–pull microdialysis procedure for antibodies is challenging and requires extensive training, recent studies have shown that it can provide direct *in vivo* measurement of free antibody concentration in selected regions of the brain in freely moving animals (Chang et al., 2018; Chang et al., 2021). Despite the expertise and experience required, this technique seems more adequate to quantify the pharmacologically active drug exposure at the site of action than quantification of total brain concentrations which represent a lumped sum of different drug exposures in the blood vessels, interstitial and intracellular fluid (Eigenmann et al., 2017a).

Quantitative insights into the physiological processes of brain uptake were provided with a reduced brain PBPK model that assumes brain uptake *via* two restricted routes, namely the BBB or the BCSFB with a minimal number of drug-related parameters that have to be estimated from the data. The model was able to well characterize the PK profiles in CSF, ISFbrain and serum of EGFRvIII-TCB after IV and ICV dosing as well as of the two additional tool compounds after IV dosing. Since we measured only local drug concentration in ISFbrain as well as CSF, the model describes net flux from brain vascular to the CSF or the ISFbrain with the corresponding estimated reflection coefficients for both compartments. The reflection coefficients can be regarded as a metric of the limited inflow of antibodies into the ISFbrain. While a reflection coefficient of 1 means that there is no antibody uptake, a reflection coefficient of 0 suggests no additional restriction. Those parameters were drug-dependent and differed between the three tool compounds.

The proposed reduced brain PBPK model considers the CSF into its largest biological subcompartments (LV, TFV, CM, and SAS) to better reflect the dosing and sampling sites, which were spatially separated as suggested by (Chang et al., 2021). Since the anticipated elimination of antibodies *via* lysosomal degradation in the brain is expected to be negligible (Eigenmann et al., 2017a) and since we did not apply a technique allowing to quantify drug elimination inside the brain, the proposed model did not include any clearance elimination pathway in CSF or brain. In addition, this was also supported with a local sensitivity analysis of the mPBPK model (Bloomingdale et al., 2021) that incorporated endosomal FcRn recycling and degradation of mAbs. Additionally, pathway analysis of local drug elimination with the mPBPK model (Bloomingdale et al., 2021) confirmed the limited impact of brain endosomal degradation on total antibody clearance, suggesting that removal of this pathway will not significantly impact the PK predictions (Supplementary Figure S3). The parameterization of the model allowed us to quantify the net brain uptake of antibodies with a minimal number of drug-dependent parameters that will be estimated. It should be noted that the total observation period of 48 h was sufficient to quantify and investigate the uptake into CSF and ISFbrain, however due to the short observation period the systemic antibody clearance values of all three compounds could not be well estimated. The model allowed quantification of the drug uptake via BBB and BCSFB barriers by estimating the parameters related to net uptake of drug through the BBB (σuptake,BBB) and BCSFB (σuptake,BCSFB). The highest uptake *via* BBB to ISFbrain was predicted for EGFRvIII-TCB, followed by DP47-IgG, which is in turn closely followed by DP47-TCB. Ruano-Salguero and Lee (Ruano-Salguero and Lee, 2020) propose that IgG transcytosis across BBB occurs by a nonspecific process originating from fluid-phase endocytosis supported by the results of an *in vitro* study investigating antibody-transcytosis across brain-endothelial cells. In addition, Chako and colleagues (Chacko et al., 2013) have described the process of “adsorptive transcytosis” of cationized monoclonal antibodies that can increase the transcellular transport across the BBB by their interaction with naturally negatively charged plasma membranes. This could possibly explain the higher brain uptake of EGFRvIII-TCB *via* this route due to a positive charge patch on the protein surface of the EGFRvIII Fab which was not found on the DP47 Fab (Supplementary Figure S4).

It has been discussed that the CSF may contribute to the drug uptake into ISFbrain (Bloomingdale et al., 2021). To our knowledge, we are the first to estimate a reflection coefficient on the transfer between CSF and ISFbrain (σCSF_ISF) providing quantitative insights to the transfer from CSF to ISFbrain. This was rendered possible due to our experimental design with both intravenous and intracerebroventricular dosing of EGFRvIII-TCB that included measurements in both CSF and ISFbrain. The high value for σCSF_ISF (0.9994) suggests that only a minor fraction of EGFRvIII-TCB in the CSF will eventually reach ISFbrain directly after ICV dosing. This finding is clearly reflected in the PK profiles of EGFRvIII-TCB, which show that direct injection into the lateral ventricle containing CSF did not improve brain exposure in the ISFbrain as compared to systemic administration. Interestingly, pathway analysis suggests that even upon direct injection into the brain CSF through ICV dosing in the lateral ventricles, the majority of antibodies will eventually reach the ISFbrain in the prefrontal cortex through the BBB. This suggests that most antibodies will first leave the CSF and enter serum, either directly through absorption at the subarachnoid villi or indirectly through entering the lymphatics at the cribriform plate (Johnston et al., 2004), before being reabsorbed into the ISFbrain. Only a smaller fraction of the antibodies (approximately 22%) will reach ISFbrain directly from CSF (Figure 5). Upon IV dosing, the fraction of drug passing the CSF-ISFbrain barrier is negligible.

It is generally accepted that free fluid transfer is possible between the CSF and ISFbrain across the ependymal cell layers in the ventricles, which distinctively lack tight junctions (Abbott, 2004). The communication between CSF and ISFbrain is thought to be important for delivery of various solutes to the parenchyma and the removal of waste products from the ISFbrain (Carare et al., 2020). However, contrasting evidence exists concerning the permeability of the CSF-ISFbrain barrier (Brinker et al., 2014) and the underlying mechanisms. Whereas short range diffusion between CSF and ISF has been observed (Hladky and Barrand, 2014) and is in line with the leakiness of the ependymal barrier (De Bock et al., 2014), this could not explain some of the findings from tracer experiments (Iliff et al., 2012; Nattie, 2013). The clearance of solutes of varying molecular weights from ISF towards CSF was indicative of the presence of bulk-flow (Nattie, 2013) and Iliff and colleagues showed an identical clearance of two radioactive-labeled proteins that had an order of magnitude size difference, which cannot be explained by diffusion alone. Additional experiments showed that markers injected into the cisterna magna would migrate along the arteries in the subarachnoid space (Iliff et al., 2012). These results provided the first evidence of a still elusive mechanism, coined the glymphatic system, which forms an important interface between the CSF in subarachnoid space, brain vasculature, and brain parenchyma (Iliff et al., 2012). The glymphatic system allows CSF to enter the brain interstitial space through paravascular transport. The process is thought to be mainly driven by bulk flow and to be responsible for mixing of CSF and ISFbrain as well for subsequent removal of this fluid. There is an ongoing debate on the exact mechanism and anatomy of the glymphatic system, and the presence of other pathways, such as an opposite perivascular transport (Brinker et al., 2014; Bacyinski et al., 2017). Evidence suggests that there is a preferential size exclusion, with large molecules largely confined to the perivascular space (Iliff et al., 2012) and large molecules injected in either the CSF or brain parenchyma unable to cross to the other side (Szentistvanyi et al., 1984; Hutchings and Weller, 1986). This seems to support our hypothesis that the transfer of antibodies is severely impeded between CSF and ISFbrain, which was suggested by the rPBPK model.

While we were able to estimate the reflection coefficient σCSF-ISF from simultaneous fitting of ICV and IV data, we could not conclude if this is a drug-dependent parameter that may also vary between the compounds. It should also be considered that the parameter estimate may be confounded by experimental conditions. CSF was sampled from the cisterna magna, which is proximal to the site of entry into the ISFbrain. ISFbrain was sampled from the prefrontal cortex. When we consider the distal location of the prefrontal cortex relative to the cisterna magna, and the multidirectional CSF flow within the subarachnoid space as well as CSF outflow at the arachnoid villi and towards the lymphatics, it is possible that part of the antibodies in SAS got diluted and cleared. Additionally, there is a high tortuosity within the brain’s extracellular space, which prevents bulk flow (Sykova and Nicholson, 2008). This implies that antibodies that enter the ISFbrain more proximal to the CM are unlikely to contribute to antibody exposure in the ISFbrain in the prefrontal cortex. In order to elucidate this pathway, ISFbrain measurements should be made at various locations along the caudo-rostral orientation of the subarachnoid space.

A limitation of our experimental design was the large injection volume required for ICV dosing, which exceeded the volume of a lateral ventricle. Although no changes in animal behavior were observed during as well as immediately after the administration and basal CSF sample collection decreased the relative volume prior to start of the infusion, it is unclear which impact the additional volume has on CSF hydrostasis and how this may influence the PK of EGFRvIII-TCB.

Lastly, a key limitation of the proposed reduced PBPK model is that is relies on physiological parameters with uncertainty in their respective values such the flow and production rates of CSF and ISF (Westerhout et al., 2012; de Lange, 2013; Yamamoto et al., 2018; Chang et al., 2019). In addition, it has been discussed that these processes may be altered with neurological disorders or physiological states (Bacyinski et al., 2017; Mestre et al., 2020); however, this was not considered with the current model. Further studies are required to investigate these and to refine the model accordingly. This will be a crucial step to enable physiological interpretation of the parameters and to enable the prediction of the anticipated brain uptake in patients with neurological disease based on the healthy status.

A recent study by Chang and colleagues (Chang et al., 2022) suggests a bell-shaped profile between antibody size and ISFbrain exposure with an optimal antibody size around 100 kDa (Chang et al., 2022). However, based on our study it remains inconclusive to what extend the size and the biophysical properties contribute to small difference in brain uptake between DP47-IgG and DP47-TCB especially given that EGFRvIII-TCB, which has the same size as DP47-TCB, is attributed with a markedly higher uptake *via* BBB. For further investigation, additional studies are proposed to analyze more compounds that differ in size and with varying biophysical properties.

### 4.1 Conclusion

In this work, we conducted a mechanistic PK study to investigate the disposition of antibody based therapeutics to the brain appling microdialysis. The PK data in CSF, ISFbrain and serum have been analyzed with a reduced brain PBPK model to assess the relative contributions of net uptake via BBB and BCSFB barriers. The modeling results suggest that there is a limited transfer of antibody directly from CSF to ISFbrain. This apparent barrier between CSF and ISFbrain could be explained by preferential size exclusion in the perivascular spaces, but is likely also the result of antibody dilution and removal from the CSF, leading to lower than expected levels of subarachnoid-parenchymal transport. This may explain why ICV dosing did not result in higher ISFbrain exposure as compared to the IV route of administration and may have implications on the choice of the preferred administration route. The proposed model provides mechanistic insights into physiological processes related to brain uptake such as the relative contributions of the blood-brain and blood-CSF barriers to the total brain uptake.

## Data Availability

The datasets presented in this study can be found in online repositories. The names of the repository/repositories and accession number(s) can be found below: https://github.com/PKPD-coder/PBPK_model_antibody_brain_uptake.git.
