# Supplementary material for: Investigating brain uptake of a non-targeting monoclonal antibody after intravenous and intracerebroventricular administration
Source: Front Pharmacol. 2022 Aug 29;13:958543. doi: 10.3389/fphar.2022.958543 (PMC9465605; doi:10.3389/fphar.2022.958543)
Supplement: Supplementary file 1 [file DataSheet1.docx]

Supplementary Material

Investigating brain uptake of a non-targeting monoclonal antibody after intravenous and intracerebroventricular administration

Arthur J. Van De Vyver, Antje-Christine Walz, Mariette S. Heins, Afsaneh Abdolzade-Bavil, Thomas E. Kraft, Inja Waldhauer, Michael B. Otteneder

Corresponding Author: antje.walz@gmx.de

# Supplementary Figures and Tables

## Supplementary Figures

**
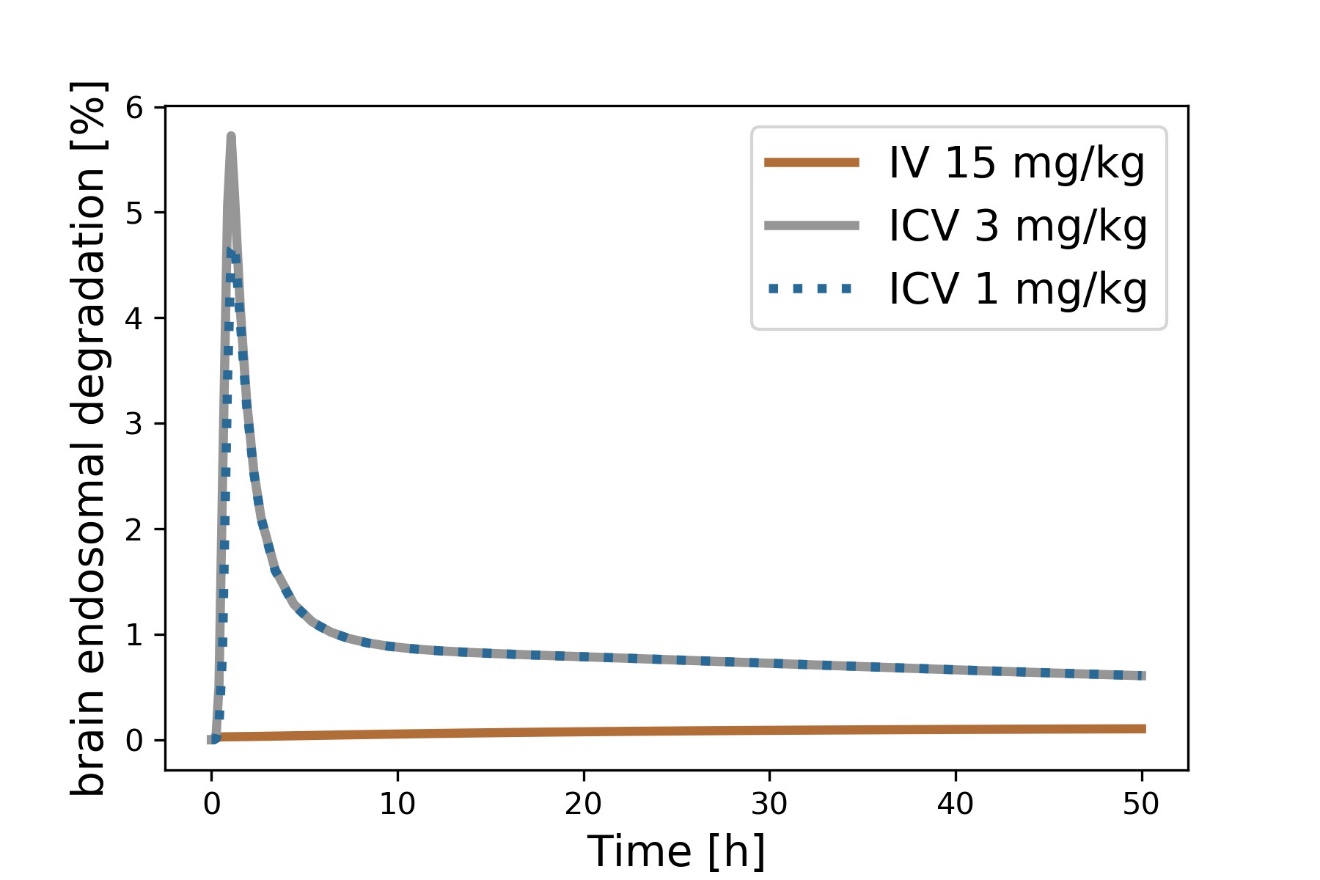
**

**Supplementary Figure S1.** Relative contribution (%) of brain endosomal degradation to the total clearance of EGFRvIII-TCB after 15 mg/kg IV dosing (orange, solid line), 3 mg/kg ICV dosing (grey, solid line), or 1 mg/kg ICV dosing (blue dotted line). The contribution of brain endosomal degradation is low and only shortly peaks early upon ICV dosing. Pathway analysis performed based on the mPBPK model from (Bloomingdale et al., 2021).


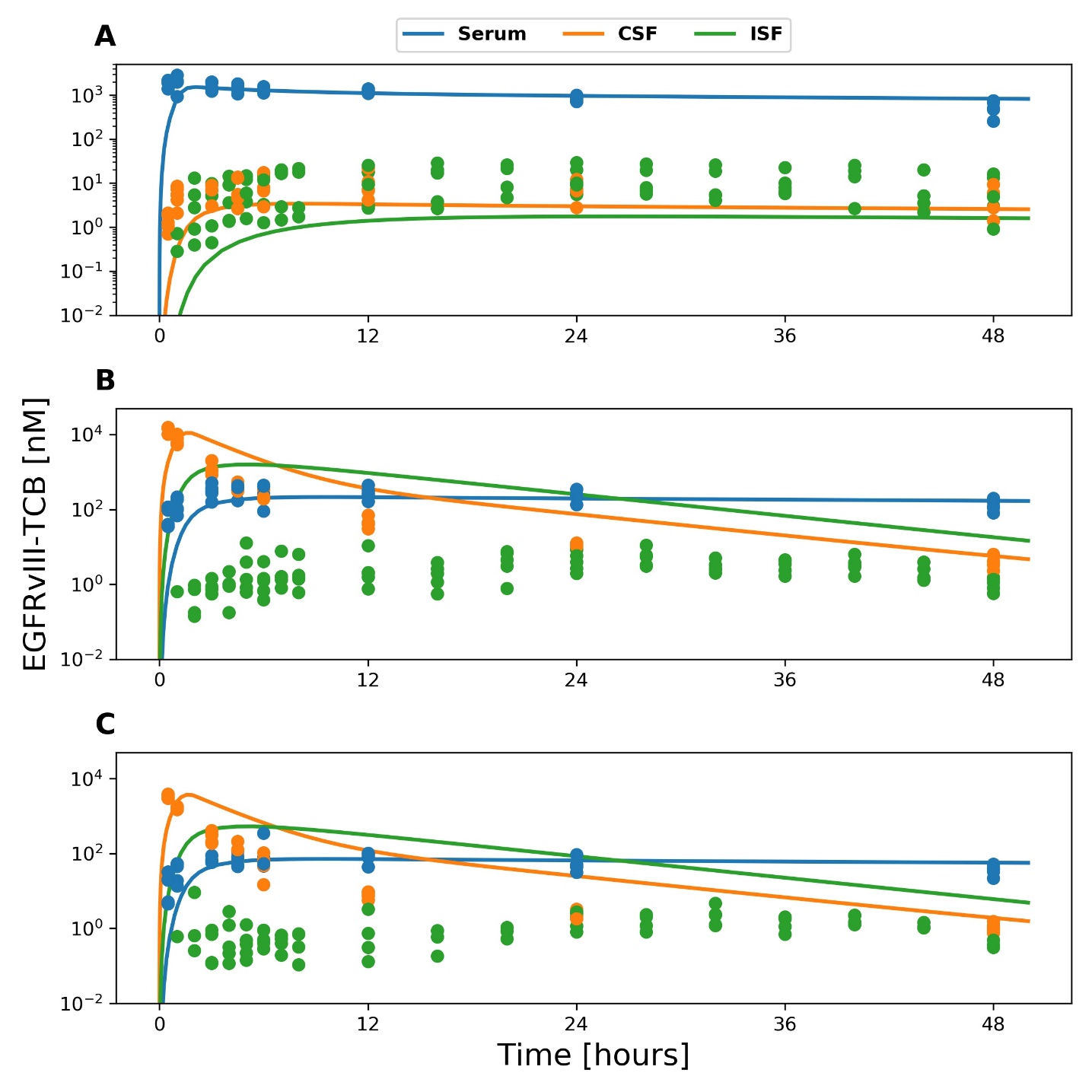


**Supplementary Figure S2.** Simulations (solid lines) of original mPBPK model overlaid with the means of observed data (symbols) for serum (blue), CSF (orange), and ISF_brain_ (green) after (A) 15 mg/kg IV dosing, (B) 3 mg/kg ICV dosing, (C) 1 mg/kg ICV dosing.


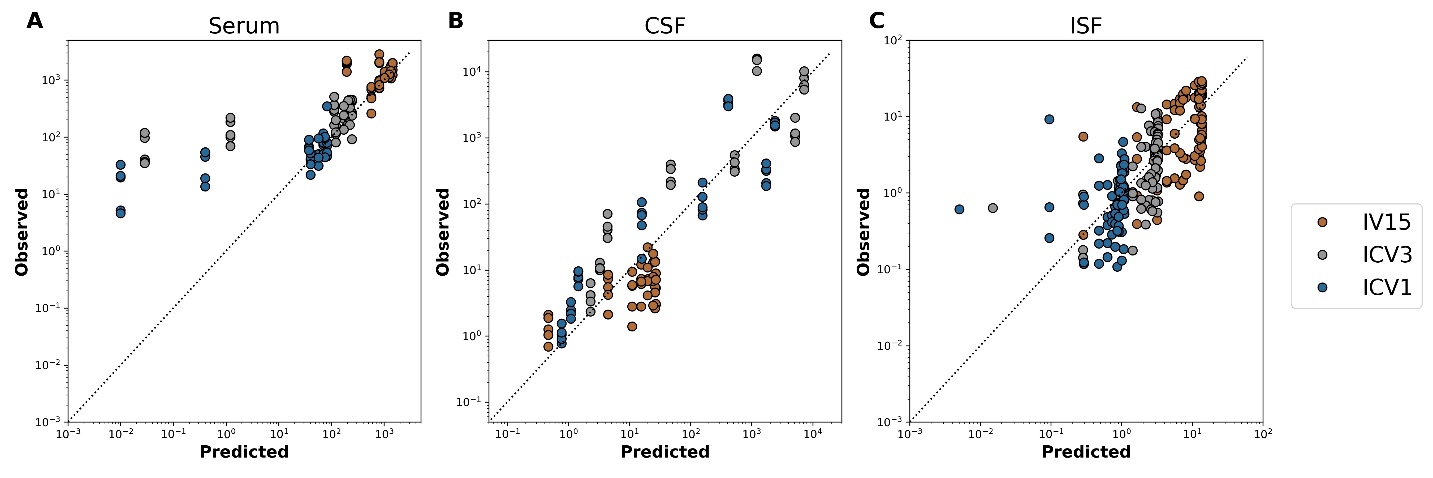


**Supplementary Figure S3.** Observed-versus-predicted plots of EGFRvIII-TCB PK in (A) Serum, (B) CSF, (C) ISF_brain_, after 15 mg/kg IV dosing (orange), 3 mg/kg ICV dosing (grey), or 1 mg/kg ICV dosing (blue).


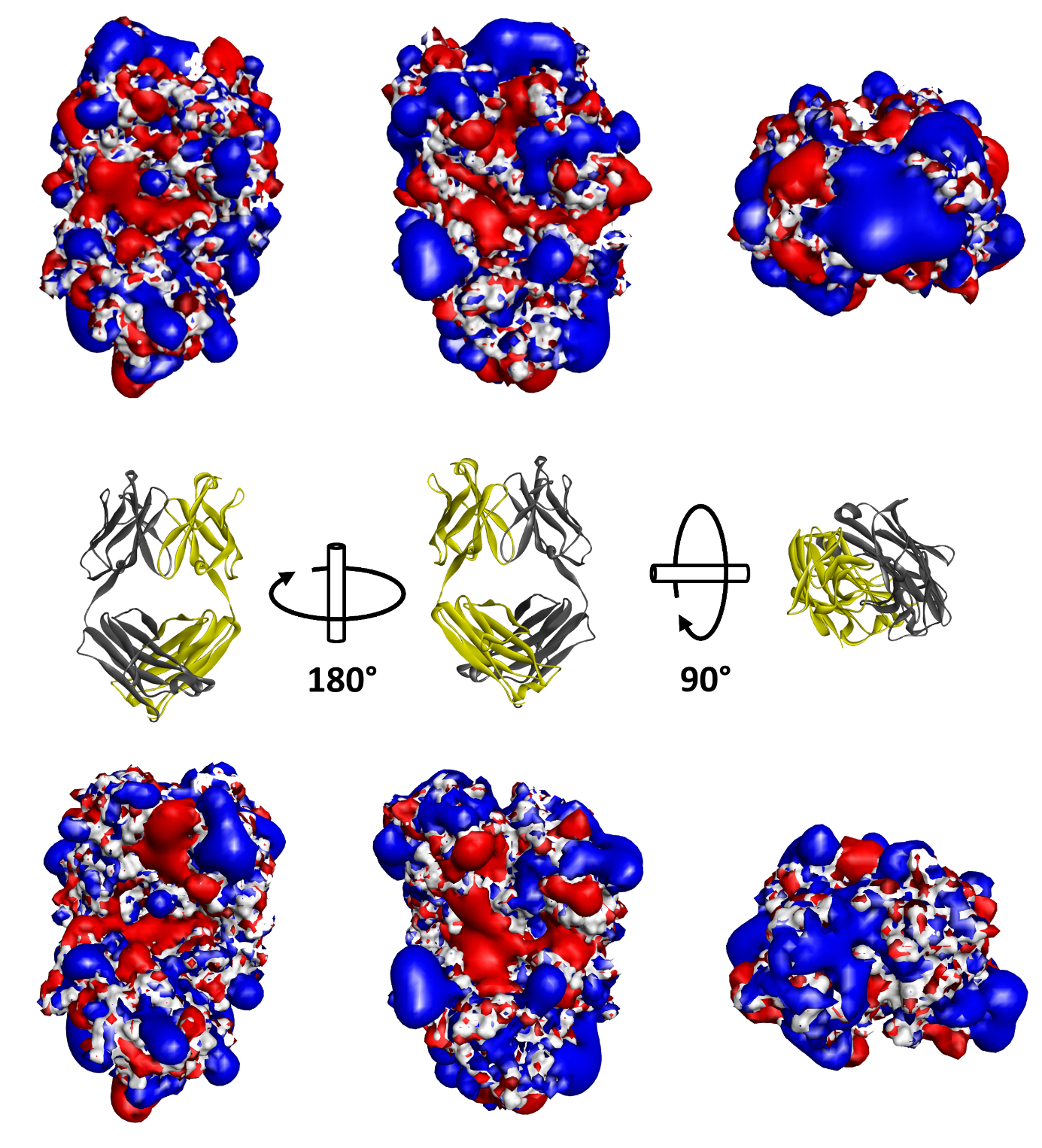


**Supplementary Figure S4.** Isopotential surface of EGFRvIII and DP47 Fabs**.** Colors indicate positive (blue), negative (red) and neutral (white) charges on Fab surface. **A:** Front, back and top view of EGFvIII Fab with large positively charged patch on top in CDR region. **B:** Ribbon diagram of generic Fab, indicating heavy chain (yellow) and light chain (grey) as well as spatial orientation of EGFRvIII and DP47 Fabs in A and C. **C:** Front, back and top view of DP47 Fab showing an even charge distribution.

## Supplementary Table

**Supplementary Table S1**. Comparison of prediction errors (PE%) of mPBPK and rPBPK models

| **Dosing regimen** | **Compartment** | **mPBPK**  **PE%** | **rPBPK**  **PE%** |
| --- | --- | --- | --- |
| 15 mg/kg IV | Serum | 34.9 | -3.3 |
|  | CSF | -47.9 | 133.2 |
|  | ISF | -83.5 | 11.5 |
| 3 mg/kg ICV | Serum | 7.3 | -19.2 |
|  | CSF | 80.0 | 32.9 |
|  | ISF | 14731.0 | -4.4 |
| 1 mg/kg ICV | Serum | 39.0 | 4.8 |
|  | CSF | 149.9 | 84.5 |
|  | ISF | 11452.7 | -25.5 |

# References

BLOOMINGDALE, P., BAKSHI, S., MAASS, C., VAN MAANEN, E., PICHARDO-ALMARZA, C., YADAV, D. B., VAN DER GRAAF, P. & MEHROTRA, N. 2021. Minimal brain PBPK model to support the preclinical and clinical development of antibody therapeutics for CNS diseases. *J Pharmacokinet Pharmacodyn,* 48**,** 861-871.
